# Cognitive-Behavioral Approaches to Alcoholism Treatment

**Published:** 1994

**Authors:** Ronald M. Kadden

**Affiliations:** Ronald M. Kadden, Ph.D., is a professor in the Department of Psychiatry, University of Connecticut School of Medicine, Farmington, Connecticut

## Abstract

Cognitive-behavioral theories explain alcoholism as a learned behavior that can be changed using the same behavior modification interventions employed to alter other learned behaviors. Treatment interventions teach clients the skills they need to confront or avoid everyday situations that may lead to drinking.

Understanding what motivates people to drink abusively involves knowing their drinking behaviors and patterns and their perceptions, or cognitions, about themselves and about alcohol. Cognitive-behavioral psychology incorporates all of these elements, expanding the basis of traditional behavior theory beyond an exclusive focus on observable behaviors to include thoughts and emotions. Although it has broadened the scope of behavior theory, this perspective still employs many behavioral techniques to help clients who have psychological problems to change. As applied to alcoholism,^1^ the cognitive-behavioral approach (see box; Kazdin 1982) views the etiology and persistence of pathological drinking as learned behavior (discussed below) and has led to the application of learning-based clinical methods to alcoholism treatment. These clinical techniques include coping skills training, relapse prevention, marital or family interventions, and development of broad-based social supports for clients. These and other related methods are described in this article, along with considerations of their effectiveness.

**Distinguishing Behavioral and Cognitive-Behavioral Theory**

Behavioral approaches deal exclusively with observable behaviors and the observable antecedents and consequences that control behavior. Behavioral approaches do not consider unobservable events, such as thoughts or emotions, that a therapist can know only through the client’s self-reports or through inferences from the client’s behavior.

Cognitive-behavioral approaches, on the other hand, freely include internal events, such as thoughts and feelings that are known only through self-reports, in conceptualizing the factors that precipitate and maintain behavior. These approaches make use of learning techniques (e.g., repeated practice, modeling, and reinforcement) to modify the client’s behavior, thoughts, and feelings.

## Overview of the Cognitive-Behavioral Theory

Cognitive-behavioral theory views alcohol dependence as a maladaptive means of coping with problems (e.g., social problems) or meeting certain needs. From this point of view, alcoholic drinking is a sequence of learned behaviors acquired in the same manner as any other learned behaviors: through imitating role models, as a result of experiencing the positive effects of alcohol (e.g., reducing anxiety, relieving pain, or enhancing sociability), or based on expectations that alcohol will have one or more of these effects (Monti et al. 1989). After repeated positive experiences with alcohol, some individuals begin to rely on alcohol consumption as the preferred way of coping with problems or meeting needs, especially because alcohol’s effects are felt fairly rapidly and require relatively little effort on the part of the drinker. According to cognitive-behavioral theory, these learned drinking patterns can be altered through the application of combined cognitive and behavior modification interventions, which can help people with alcohol dependence achieve and maintain sobriety (Miller and Hester 1989).

In addition to the view of alcoholism as a learned phenomenon, other theories have been developed to account for its origins. One of these focuses on genetics as a factor in the development of alcohol dependence. Even from this perspective, however, the way in which a genetic vulnerability is expressed is dependent upon a person’s psychosocial experiences (Tarter and Vanyukov 1994). As a result, whatever drinking patterns are acquired ought to be amenable to remediation by cognitive-behavioral interventions (Monti et al. 1989).

### Antecedents of Drinking Alcohol

The cognitive-behavioral approach to alcoholism treatment focuses on the factors that precipitate and sustain drinking. Antecedents are the events that occur prior to drinking and either set the occasion for it or initiate a chain of behaviors that culminates in drinking. Different types of antecedent factors may lead to a person’s drinking (Miller and Mastria 1977). They can be social, such as interpersonal conflict or peer pressure; environmental, such as alcohol advertisements or seeing others drink; emotional, such as anger or depression; cognitive, such as negative thoughts about oneself; and physiological, such as chronic pain or withdrawal symptoms. The focus of cognitive-behavioral treatment is on identifying the most common and most potent antecedents for each client and breaking their connection with alcohol use by training the client to respond to them in new ways.

### Consequences That Reinforce Drinking

Other factors may contribute to a client’s alcoholism by maintaining the drinking behavior pattern after it has begun; these include various positive consequences that reinforce drinking. Like the antecedents, consequences can be grouped into several categories: social, such as praise from friends; emotional, such as reduced anxiety or enhanced emotional expression; cognitive, such as increased positive thoughts about oneself; and physiological, such as decreased pain or reduced withdrawal symptoms (Miller and Mastria 1977).

Cognitive-behavioral treatment addresses these events that occur after drinking. It involves arranging alternative, less desirable consequences for drinking (e.g., a significant other withdrawing from social interactions with the drinker) and arranging positive consequences for sobriety (e.g., a significant other providing attention and recognition).

### Behavioral Deficits That Impede Treatment

One complication in treating those with alcohol problems can be the clients’ inability to implement the changes specified in a treatment plan because of their behavioral deficits. That is, these clients may never have acquired the appropriate coping behaviors or skills, or, having learned them at one time, they cannot retrieve the skills either from lack of practice or from some inhibition. Whatever the reason for them, behavioral deficits are considered to be a significant obstacle to recovery from alcoholism (Miller and Hester 1989). For clients with these deficits, coping skills training (described below) often is necessary to teach sufficient or refresh deficient coping behaviors, reduce any inhibiting factors that might prevent their use, and provide practice so that the skills will be readily available when needed.

## Coping Skills Training

The coping skills training approach to alcoholism treatment addresses a client’s behavioral deficits, responses to antecedents, and the consequences of drinking. Training is based primarily on principles of social learning theory, which considers cognitive, environmental, and behavioral factors (Abrams and Niaura 1987). The emphasis is on teaching or enhancing the skills required for achieving abstinence and for coping with the problems of daily life that could lead to drinking.

The first step in this process is a functional analysis of the client’s drinking behaviors to determine the relationships between drinking and its antecedents and consequences. Knowledge of these relationships helps to clarify drinking’s role in the client’s life and provides a focus for efforts to change behavior. Although clients rarely can articulate exactly what causes them to drink, they often are able to identify personal or environmental events that tend to precede or follow their drinking. The therapist can obtain this information through a clinical interview or by using structured assessment instruments, such as the Inventory of Drinking Situations (IDS) (Annis and Davis 1989) discussed below. (For a description of additional assessment instruments and methods, see Donovan and Marlatt 1988.)

Once the client’s drinking pattern and the influences that support it have been clarified, the therapist can identify the skills the client must learn to alter the chain(s) of events leading to drinking. These skills also will help the client address the consequences if drinking occurs. Monti and colleagues (1989) have characterized the skills that must be taught as either intrapersonal or interpersonal and have developed a session-by-session manual for implementing a comprehensive skills training program (table 1). Skills are described in more detail below based on material presented in that treatment manual.

### Intrapersonal and Interpersonal Skills Training

#### Intrapersonal Skills

The therapist’s functional analysis of the client’s drinking patterns may identify internal events that play a role in drinking. Some events are likely to be alcohol related, such as thoughts of alcohol and outright cravings, and clients must be taught skills to respond to them effectively. For example, clients are encouraged to develop a list of activities they could engage in to distract themselves until a craving passes. They also are given practice in challenging their thoughts about drinking by reviewing the risks entailed and considering alternative ways to respond to these thoughts. Clients routinely may have tended to resolve problem situations, even when they were unrelated to drinking, in ways that automatically resulted in drinking. Training in problem-solving and decisionmaking skills can improve the clients’ ability to cope more effectively and anticipate the consequences of their actions.

Clients’ coping skills also may be improved by stress management training, a treatment component that can include relaxation training (e.g., using slow breathing to relax and control anxiety), systematic desensitization (e.g., learning to tolerate feared situations through gradual exposure to them), and cognitive strategies (e.g., correcting negative interpretations of events) (Stockwell and Town 1989). Other intrapersonal skills that may be taught include coping with anger or with negative thoughts (see Kadden et al. [1992] for techniques related to coping with depressed moods).

Therapists also may have to help their clients fill the leisure time they gain when they stop drinking. Unless free time is used appropriately, it could lead to boredom and attendant cravings for alcohol or to activities that increase the chances of drinking. Thus, clients should sample various leisure activities to find those that they enjoy, that are incompatible with drinking, and that also could be used from time to time as rewards for specific accomplishments along the road to sobriety.

Finally, it is unlikely that therapists will be able to identify all the factors relevant to a client’s drinking or anticipate all possible high-risk situations. Therefore, each client should develop a set of emergency plans for confronting any unforeseen situations that may arise. These plans usually include coping skills developed over the course of treatment, such as managing thoughts about alcohol, developing problem-solving skills, and telephoning people in one’s social support network. In addition, clients need to formulate plans for coping with persistent problems that cannot be resolved and are likely to present continuing challenges to sobriety.

#### Interpersonal Skills

In many cases, the therapist’s functional analysis of the client’s drinking behaviors identifies problems encountered in interactions with others. Consequently, clients have to learn to resist offers to drink or related forms of social pressure from coworkers, friends, or even family members. In addition, clients may be deficient in very basic social skills, leaving them isolated and without adequate social support, which are common antecedents to drinking. These clients benefit from training in starting conversations, nonverbal communication (body language), giving compliments, being assertive, refusing requests to do things for others that will overburden them, communicating emotions, and improving functioning in an intimate relationship. Clients also can learn to handle criticism so that neither giving it nor receiving it will arouse strong negative emotions that could lead to a relapse. Finally, training may be necessary in the development and nurturance of a social support network, which would enhance the likelihood of their maintaining sobriety.

### Training Influences

The cognitive-behavioral approach concerns not only the content of therapy but also the manner in which it is delivered (Monti et al. 1989). Verbal descriptions of new skills are kept to a minimum, with the therapist placing greater emphasis on modeling the skills and on active practice by clients. Practice should include a wide variety of role-play scenarios to broaden the skills’ applicability. Likewise, homework assignments may be given to foster use of the skills in real-life situations. Frequent reviews of previously taught skills will enhance the clients’ mastery and help to counter problems they may have in retaining the skills.

#### Sequence of Skills Training

Although good pedagogy suggests that learning should proceed from the simplest skills to the more complex, some treatment situations require that therapists first provide training in complex skills, which are essential for abstinence, to prevent relapse and early dropout from treatment. Clients who live at home and receive outpatient treatment, for example, are likely to encounter high-risk situations daily that require complex skills. It often is necessary for therapists to teach their clients how to manage their anger or how to manage their thoughts about drinking prior to teaching more basic skills, such as starting conversations or nonverbal communication.

#### Influence of the Treatment Setting

Methods of cognitive-behavioral skills training also may be influenced by whether therapy takes place in an individual or group context. In coping skills training, group therapy provides a convenient setting for skills modeling, rehearsal, and feedback, and it allows clients to share experiences they have had using the skills that they are being taught. Individual therapy provides greater attention to people for whom group therapy may not be recommended (indications for individual therapy are reviewed by Rounsaville and Carroll 1992). In this case, the therapist assumes functions usually assigned to group members.

Organized inpatient and partial hospital treatment programs may use systems of rewards and penalties, called contingency management techniques, to reinforce appropriate client behaviors. Such appropriate behaviors can include participating in treatment activities, practicing skills, and planning for continued care after discharge from the program. Contingency management may be useful particularly for clients who are impulsive, who require structure, or who may be poorly motivated.

Clients who participate in inpatient and partial hospital treatment programs receive a list of routines and rules that specify consequences for infractions and rewards for appropriate behaviors or successful completion of program goals. Clients may select rewards from options ordinarily available in the program environment, such as freedom to move about the facility unescorted, off-grounds passes, unlimited telephone privileges, gifts of literature from Alcoholics Anonymous, and a certificate upon completion of treatment. Negative consequences for infractions of rules may entail a loss of certain privileges (for further detail, see Kadden and Mauriello 1991).

## Relapse Prevention

This approach to treatment is similar to coping skills training and employs many of its methods. Relapse prevention, however, has fostered a closer analysis of the relapse process, focusing on interventions that may be used to interrupt it and identifying behaviors that should be strengthened to maintain long-term sobriety.

**Figure 1:**
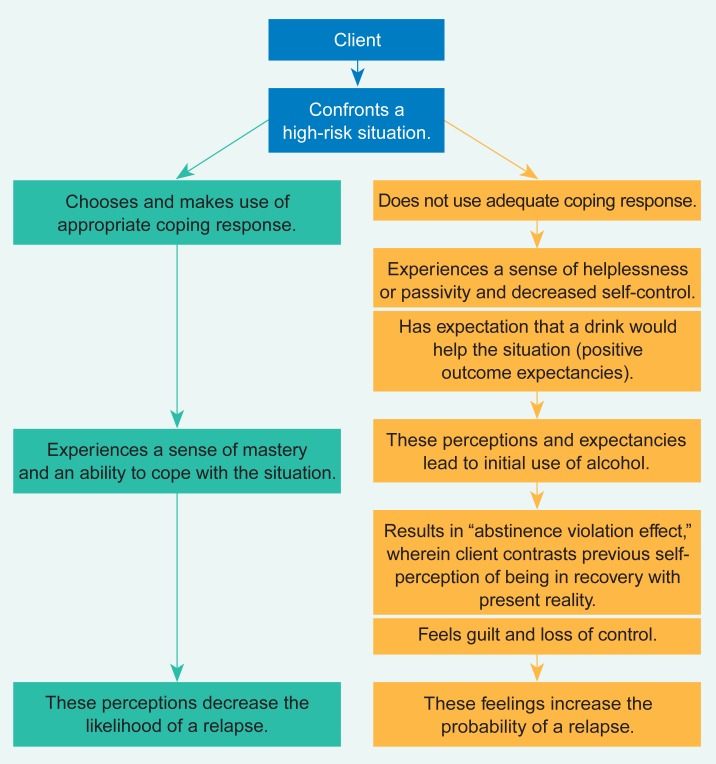
A cognitive-behavioral model of the relapse process. SOURCE: Adapted from Marlatt 1985.

Figure 1 provides Marlatt’s (1985) schematic of the relapse process, depicting two possible responses to a high-risk situation. As shown, when clients choose and execute an appropriate coping response, they feel a sense of mastery, but when no coping response is used, they feel helpless and anticipate that a drink would help in the situation. Such thoughts are likely to be followed by drinking, and the clients then contrast the previous perception of being in recovery with the current reality of renewed drinking. This incongruity leads to feelings of conflict, guilt, self-blame, and perceived loss of control, a syndrome that Marlatt has called the “abstinence violation effect.” Because of this despair, further drinking becomes likely and often leads to a full-blown relapse (Marlatt 1985).

The therapist may use several of the following interventions to help clients avoid high-risk situations (Marlatt and Gordon 1985):

- Modifying clients’ lifestyles by strengthening behaviors that are incompatible with drinking
- Providing training in decisionmaking and self-control that enables clients to make appropriate choices
- Establishing a balance between the time clients spend meeting responsibilities and the time spent on pleasurable activities.

Central to this approach is the enhancement of clients’ awareness of high-risk situations when they are at an early stage, during which the situations do not appear overwhelming and therefore are easiest to manage. Therapists teach clients to monitor and evaluate their feelings, their thoughts, and the situations in which they find themselves to identify potential antecedents to drinking. Successful coping with these antecedents requires that clients acquire skills for managing external triggers, handling their emotions, and countering cognitive distortions about themselves.

Clients’ expectancies regarding the positive effects of alcohol can be combated if the therapist educates the clients about the delayed negative effects of drinking and suggests that they carry a reminder card listing the negative effects they have experienced. Clients may be taught to view a slip (e.g., taking a drink) as a learning experience and an opportunity to formulate more effective plans for coping with similar situations in the future.

The IDS, developed by Annis and Davis (1989), offers a systematic way of evaluating relapse risk that can be helpful in treatment planning. The IDS is a detailed assessment instrument that determines clients’ specific high-risk situations and provides profiles of typical situations that are likely to pose problems. Annis and Davis also emphasize the importance of assessing clients’ strengths and available resources, in addition to risks, to determine the most appropriate starting point for a skills training program.

## Behavioral Marital and Family Therapy

Family members seem to be well positioned to support a client’s recovery process. They may, however, have little knowledge about alcohol dependence, may be misinformed about how to respond to their loved one’s condition, and may have developed troublesome behavior patterns of their own that could sabotage the client’s recovery. Family members generally require education about alcohol and its effects as well as opportunities to discuss the impact that a loved one’s alcoholism has had on their lives.

Coping skills training approaches have been developed for alcoholic couples (i.e., behavioral marital therapy; O’Farrell and Cutter 1984) and have been broadened for use with other family members as well (O’Farrell and Cowles 1989). Interventions include training in communication skills, conflict resolution, and problem-solving; breaking the cycle of criticism and recriminations; providing mutual praise for positive changes; and discovering shared leisure activities. To help structure their new interactions, couples and families can be helped to formulate behavior contracts that clearly specify each person’s role. (For more complete descriptions and case examples, see O’Farrell and Cowles 1989 and McCrady 1985.)

## Community Reinforcement

This treatment approach incorporates various behavioral procedures into a comprehensive intervention package, with special emphasis on clients’ making use of community-based supports (Sisson and Azrin 1989). Its chief goal is to develop a highly reinforcing sober lifestyle that clients will seek to perpetuate, thus addressing not only the drinking problem but also the negative lifestyle factors likely to undermine recovery. With such a positive lifestyle in place, drinking likely will be avoided, because it would result in the withdrawal of valued social supports and reinforcers. This approach thus may be considered a form of contingency management (discussed above).

The community reinforcement approach combines several interventions. Recognizing the powerful role of social contingencies in the recovery process, this approach provides social skills training; a “buddy,” whose role is to support the client’s efforts at maintaining sobriety; counseling regarding leisure and recreational activities; and an alcohol-free social club that sponsors activities and provides a safe place for clients to “hang out.” Unemployed clients are referred to a supportive “job club,” where they can organize their résumés, learn about job leads, and practice interview techniques. Clients also are taught strategies for coping with urges to drink and refusing offers to drink. They are encouraged to take disulfiram (Antabuse^®^) as a deterrent to drinking and to identify a significant other who will support them in taking the medication each day. Finally, clients are offered behavioral marital counseling as a means of reducing stress at home, improving communications within the family, and enhancing support for sobriety.

Provision of this wide range of services requires that several specialized program elements be in place and necessitates considerable organization on the part of treatment staff to ensure that each client receives a coordinated package of services that will meet his or her particular needs.

## Behavioral Self-Control Training

This variant of the cognitive-behavioral approach focuses on what clients can do to modify their own behavior. It includes identifying drinking situations, setting goals, monitoring oneself, learning and practicing coping skills, and rewarding oneself for accomplishing goals (Hester and Miller 1989). Clients can receive guidance from a therapist or through the use of a self-help manual. In either case, the client assumes responsibility for determining the content and pace of treatment. Self-control training may have a goal of total abstinence, but more often it uses a goal of “controlled drinking” for clients who have shorter durations of problem drinking and relatively few alcohol-related problems. This goal is not used for heavily dependent drinkers.

## Aversion Therapy

This approach seeks to develop a conditioned aversion in the client by associating an aversive event with alcohol. Treatment involves either pairing stressful or painful stimuli (e.g., nausea or electric shock) with actual alcohol consumption or pairing images of drinking with images of unpleasant scenes or experiences. Effectiveness of the procedures is enhanced when they are combined with other cognitive-behavioral strategies (Rimmele et al. 1989). Aversion therapies have been implemented in only a few treatment centers and have not been adopted widely by treatment providers.

## Cue Exposure Therapy

Cue exposure therapy seeks to diminish a client’s responsiveness to antecedent factors that lead to drinking (Cooney et al. 1983). It involves repeatedly presenting a client’s favorite alcoholic beverage, encouraging the client to observe and smell the drink, but not allowing the client to consume any of it. The arousal generated in this situation may be heightened by having the client simultaneously imagine an emotional scene in which he or she would be likely to drink. The repeated exposures to alcohol, without the reinforcement of actual drinking, may reduce its power either to elicit cravings or to signal an opportunity to drink. This treatment also may provide an opportunity to practice coping skills in the presence of alcohol (Monti et al. 1989). Cue exposure therapy is still in the experimental stage, with support for its efficacy thus far coming mainly from case reports (Institute of Medicine 1990).

## Motivational Interviewing

Although not strictly a cognitive-behavioral approach, this technique is included here because it incorporates behavioral procedures, such as shaping and reinforcing clients’ statements about the need for change, and has as a goal the development of strategies for changing behavior.

Poor motivation for change is an age-old problem, particularly in the field of alcoholism treatment, where clients’ ambivalence has led to a troublesome lack of treatment compliance. Recently, a systematic approach called motivational interviewing has been developed to enhance client motivation. It is based on principles of cognitive therapy and the client-centered approach developed by the psychologist Carl Rogers (Miller and Rollnick 1991). Its goal is to help clients resolve their ambivalence and reach a commitment to change.

Motivational interviewing starts with the therapist recognizing and accepting client ambivalence. Proceeding through what may be characterized as a gradual shaping process, the therapist tries to move the client toward acknowledging current problems, developing a desire to change them, and identifying strategies that will enable this change.

The therapist first discusses problems that the client has perceived or concerns that others have voiced, providing empathic feedback, which communicates an understanding and acceptance of the client. These interventions attempt to establish a climate in which the client feels safe enough to identify and explore areas of dissatisfaction with his or her life. Using this process, the therapist avoids arguing with the client, confronting the client’s resistance head on, or labeling the client as an alcoholic. The therapist instead assumes a reflective attitude to allow exploration of both sides of the client’s ambivalence without unduly arousing defensiveness. Throughout the course of the discussion, the therapist provides frequent summaries of what the client has said to focus attention on the problems that are being uncovered and to highlight whatever motivational statements the client has made along the way.

Through a gradual application of this process, the client is made increasingly aware of problems that he or she may have been ignoring; through this awareness, the client is brought to the point of accepting the need for change and then to formulating a strategy for making behavior changes. Subsequent checkup visits may be used to maintain the client’s motivation and to determine whether the client has followed through with agreed-upon change strategies.

## Effectiveness of Cognitive-Behavioral Approaches

Cognitive-behavioral approaches to alcoholism were developed from behavior change principles that have been applied to a wide range of disorders, and their application to alcohol problems has been guided by empirical research findings (George and Marlatt 1983; Abrams and Niaura 1987). Such findings support the effectiveness of these treatments in addressing several problems that alcoholics typically confront as they seek to recover (e.g., coping with high-risk situations).

In addition, other considerations support the use of cognitive-behavioral treatment methods for alcoholic clients, specifically in the early stages of their recovery from alcohol dependence. Even after they have completed detoxification, for example, clients may experience persistent cognitive impairment, such as faulty memory, from the effects of alcohol. The impairment often coincides with the time during which treatment is given. In view of such impairment, it has been suggested (e.g., Goldman 1987) that clients would benefit from a structured treatment approach, such as one of those reviewed here, that breaks learning tasks into small, easily mastered units and provides repeated practice and review of new skills to enhance retention (although not all research supports this recommendation).

Another consideration is put forward by some advocates of dynamically oriented therapies that seek to develop clients’ insight through the exploration of conflicts and other sources of emotional distress. There is concern that the discomfort typically associated with uncovering longstanding psychological problems and emotional material may increase the probability of relapse and therefore should be avoided in the early stages of recovery (Zweben 1986). An intervention that focuses on concrete tasks (as cognitive-behavioral approaches do), rather than on exploring emotional matters, avoids this problem and may be the treatment of choice for many clients early in recovery.

### Results of Treatment Outcome Studies

In a comprehensive review of research on alcoholism treatment outcome, Miller and Hester (1986) identified social skills training, stress management, and the community reinforcement approach as receiving sound support from controlled studies that have been replicated. The clients who benefited most from these approaches had skills deficits in areas specifically addressed by the treatment they received. Another review of alcoholism treatment effectiveness, conducted by the Institute of Medicine (1990), cited social skills training, marital and family therapy, stress management training, and the community reinforcement approach as showing “promise for promoting and prolonging sobriety” (p. 538). The same report also noted that behavioral self-control training appears effective for clients who are not severely alcohol dependent.

Two of the interventions described above have not yet been widely used or extensively tested, although promising outcomes have been reported. Studies of motivational interviewing, which is a relatively new technique, have provided early indications of effectiveness (Bien et al. 1993). Cue exposure therapy is still in the experimental stages and is not ready for widespread clinical application.

### Cost-Effectiveness

Cognitive-behavioral treatments have scientific origins and have maintained a tradition of empirical validation of clinical procedures. This scientific examination of treatment techniques may turn out to be an important advantage in the emerging climate wherein third-party payers scrutinize treatment outcome and cost-effectiveness. In a review of the effectiveness and cost-effectiveness of various treatments for alcoholism (Holder et al. 1991), those treatments with the best evidence of effectiveness were cognitive-behavioral and behavioral approaches, including social skills training, self-control training, behavioral marital therapy, the community reinforcement approach, stress management, and motivational interviewing. These also were rated as the most cost-effective, falling in a range from minimal to medium-low on a scale of costliness. Aversion therapies, on the other hand, showed either fair evidence of effectiveness (when using imagined aversive scenes) or no evidence of effectiveness (when using electric shock or nausea).

## Summary

In a cognitive-behavioral conceptualization of alcohol dependence, drinking is regarded as a learned behavior that can be altered by identifying its antecedents and consequences and by modifying the drinker’s responses to them. The treatment approaches described in this article are modeled in a variety of ways on this basic precept. With the possible exceptions of aversion therapy and cue exposure therapy, these various approaches have been found to be both effective and cost-effective. However, because no single approach has been found effective for most alcoholics, patient-treatment matching (see sidebar) has received increased attention as a way of improving treatment effectiveness.

**Patient-Treatment Matching Findings**

To date, treatment outcome research has failed to identify any single approach that is superior across the varied spectrum of alcoholic clients. As a result, the alcoholism treatment field is looking increasingly to patient-treatment matching research to identify treatment approaches that will provide the most benefit to subgroups of clients with particular needs (for a more detailed review of patient-treatment matching, see the article by Mattson, pp. 287–295).

The patient-treatment matching literature is still in its infancy, with relatively few empirical studies. Nevertheless, some reports identify client characteristics indicating which clients could benefit most from what cognitive-behavioral and behavioral approaches. In one such series of reports, Kadden and colleagues (1989, 1992; Cooney et al. 1991) found that clients who had more sociopathic characteristics, more evidence of psychopathology, and a greater urge to drink (in a role-play situation) were more likely to remain abstinent and less likely to suffer renewed alcohol-related problems if they were given coping skills therapy in group treatment. (Some of these findings were replicated by Longabaugh et al. 1994.) Clients who were relatively free of these characteristics at the beginning of treatment fared better if they were assigned to interactional group therapy, a therapy that fosters insight and healthier interpersonal functioning in patients by developing a group that encourages self-disclosure and free expression of emotions.

Findings from other matching studies that employed cognitive-behavioral treatments (see review by Mattson et al. 1994) indicated the following:

- Clients with an external locus of control (i.e., those who believe that the course of their lives is determined by external forces) experience better outcomes with coping skills counseling.
- Clients who are less educated, have substantial urges to drink, or experience high anxiety benefit most from communication skills training.
- Clients who can identify specific high-risk situations do better with relapse prevention treatment.
- Single men benefit most from the community reinforcement approach.
- Clients with poor motivation benefit more from motivational interviewing than from skills-based counseling.

The emerging literature has thus identified several client characteristics that potentially could serve as the basis for matching clients to cognitive-behavioral treatments, but more work is needed to determine their practicality in clinical settings. The National Institute on Alcohol Abuse and Alcoholism is sponsoring an ongoing multisite cooperative study (Project MATCH Research Group 1993) that will have sufficient participants to test numerous hypotheses for matching clients to cognitive-behavioral treatment as well as to 12-step and motivational enhancement treatments. It is anticipated that the matching strategy will enhance the effectiveness of all treatments, including the behavioral ones, by directing their application to the clients who are most likely to benefit from them.

— *Ronald M. Kadden*

ReferencesCooneyNLKaddenRMLittMDGetterHMatching alcoholics to coping skills or interactional therapies: Two-year follow-up resultsJournal of Consulting and Clinical Psychology595986011991165584710.1037//0022-006x.59.4.598KaddenRMCooneyNLGetterHLittMDMatching alcoholics to coping skills or interactional therapies: Posttreatment resultsJournal of Consulting and Clinical Psychology576987041989255736410.1037//0022-006x.57.6.698KaddenRMLittMDCooneyNLBusherDARelationship between role-play measures of coping skills and alcoholism treatment outcomeAddictive Behaviors174254371992133243310.1016/0306-4603(92)90003-eLongabaughRRubinAMalloyPBeattieMCliffordPRNoelNDrinking outcomes of alcohol abusers diagnosed as antisocial personality disorderAlcoholism: Clinical and Experimental Research18778785199410.1111/j.1530-0277.1994.tb00040.x7978086MattsonMEAllenJPLongabaughRNicklessCJConnorsGJKaddenRMA chronological review of empirical studies matching alcoholic clients to treatmentDonovanDMattsonMJournal of Studies on AlcoholSupp 121994162910.15288/jsas.1994.s12.167722993Project MATCH Research GroupProject MATCH: Rationale and methods for a multisite clinical trial matching alcoholic patients to treatmentAlcoholism: Clinical and Experimental Research17611301145199310.1111/j.1530-0277.1993.tb05219.x8116822

## Figures and Tables

**Table 1:** Intrapersonal and Interpersonal Skills Training Elements

Intrapersonal Skills
Managing thoughts about alcohol
Problem-solving
Decisionmaking
Relaxation training
Becoming aware of anger
Managing anger
Becoming aware of negative thinking
Managing negative thinking
Increasing pleasant activities
Planning for emergencies
Coping with persistent problems

**Interpersonal Skills**

Refusing offers to drink
Starting conversations
Using body language
Giving and receiving compliments
Assertiveness training
Refusing requests
Communicating emotions
Communicating in intimate relationships
Giving criticism
Receiving criticism
Receiving criticism about drinking
Enhancing social support networks

SOURCE: Adapted from Monti et al. 1989.

## Footnotes

1 Terms such as “alcoholism” and “alcohol dependence” are understood in this article as referring to criteria for alcohol dependence and abuse in the American Psychiatric Association’s *Diagnostic and Statistical Manual of Mental Disorders, Fourth Edition*. Pathological drinking refers to the entire range of alcohol problems from mild to severe.
